# Activation of CD8⁺ T Cells in the Human *Ex Vivo* Lung Tumor Microenvironment Using Anti‐CD3/CD28 and Nivolumab

**DOI:** 10.1002/eji.70060

**Published:** 2025-09-15

**Authors:** Tonia Bargmann, Sebastian Konzok, Renato Liguori, Maximilian Fuchs, Charline Sommer, Dirk Schaudien, Charlotte Schob, Stephan Halle, Christopher Werlein, Patrick Zardo, Lavinia Neubert, Danny Jonigk, Hans‐Gerd Fieguth, Fulvia Ferrazzi, Katherina Sewald, Susann Dehmel, Armin Braun

**Affiliations:** ^1^ Fraunhofer Institute for Toxicology and Experimental Medicine, Preclinical Pharmacology and Toxicology Member of the Fraunhofer Cluster of Excellence Immune‐Mediated Diseases CIMD Hannover Germany; ^2^ German Center for Lung Research (DZL) BREATH Hanover Hanover Germany; ^3^ Department of Nephropathology, Institute of Pathology Friedrich‐Alexander‐Universität Erlangen‐Nürnberg Erlangen Germany; ^4^ Institute of Pathology Friedrich‐Alexander‐Universität Erlangen‐Nürnberg Erlangen Germany; ^5^ Hannover Medical School Institute of Clinical Chemistry and Laboratory Medicine Hannover Germany; ^6^ Hannover Medical School Institute of Immunology Hannover Germany; ^7^ Hannover Medical School Institute of Pathology Hannover Germany; ^8^ Hannover Medical School, Department of Cardiac Thoracic, Transplantation and Vascular Surgery Hannover Germany; ^9^ Institute of Pathology RWTH Aachen University Medical Faculty Aachen Germany; ^10^ Klinikum Siloah and Klinikum, Nordstadt Pathology Hannover Germany

**Keywords:** CD8^+^ T cell responses, *ex vivo* models, immunotherapy, lung cancer, patient‐derived, tumor lung slices, tumor microenvironment, tumor border

## Abstract

Despite advancements in immunotherapies, the diversity of the tumor microenvironment remains a challenge for cancer treatment. To elucidate microenvironment‐specific differences in antitumor responses, we established patient‐derived *ex vivo* tumor‐lung slices. We analyzed immune activation profiles after treatment with anti‐CD3/CD28 and the checkpoint inhibitor Nivolumab. Lung slices from non‐tumor, tumor‐adjacent, tumor‐border, and tumor‐central tissue were generated and assessed for viability, cell composition, and immune competence via flow cytometry, soluble factor secretion, and bulk RNA‐sequencing. The tumor‐border contained the highest number of immune cells (8.3‐fold vs. non‐tumor), secreted tumor markers (S100 and CA15‐3), and exhibited high levels of inflammatory mediators (IFNγ, IL‐6, and IL‐2). Treatment with anti‐CD3/CD28 increased the frequency of CD137^+^/CD8^+^ T cells and induced cytokine responses dominated by IFNγ, IL‐2, and Granzyme B. While both non‐tumor and tumor‐border tissue responded to anti‐CD3/CD28, the intensities of immune responses were highly varied. Notably, treatment with Nivolumab induced an inflammatory response primarily in the tumor‐border evidenced by IFNγ, IL‐2, and Perforin secretion alongside increased expression of CD107a on CD8^+^ T cells, in a donor‐dependent manner. Taken together, these data demonstrate how tumor‐border tissue slices can be utilized to study T cell responses in the context of the patient‐specific tumor microenvironment.

## Graphical abstract

Patient heterogeneity remains a challenge in the treatment of lung cancer. Here, patient‐derived, *ex vivo* tumor lung slices originating from the tumor‐border are used to unravel microenvironment‐specific responses to immunotherapies. The focus lies on the impact of the native tumor microenvironment on CD8^+^ T cell activation after anti‐CD3/CD28 or anti‐PD‐1 treatment.

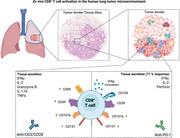

## 1 Introduction

One of the greatest challenges in treating solid tumors lies in their heterogeneous nature, which results in varied responses to immunotherapies. The success of immunotherapies in solid tumors is critically dependent on the individual immune cell composition of the patient's tumor microenvironment [1]. Consequently, there is a need to understand the response to immunotherapies within the context of the patient's specific immune landscape.

Immunotherapies like PD‐1 and PD‐L1 inhibitors have revolutionized treatment for advanced lung cancer and are increasingly used to treat lung metastases [2, 3]. Tumors with a high frequency of infiltrating lymphocytes that express proinflammatory markers are promising targets for immunotherapeutic approaches. Amongst tumor‐infiltrating lymphocytes, both the antigen specificity and activation state of the T cells play a crucial role in their antitumor activity. Tumor‐reactive T cells can be identified by expression of surface markers such as CD137, CD39, and PD‐1 [4, 5]. Furthermore, CD39^+^/CD103^+^/CD8^+^ tissue‐resident T cells have been shown to be tumor‐specific and predict the outcome of immune checkpoint blockade [6, 7, 8]. However, prolonged expression of PD‐1, CD39, and TIM3 also marks exhausted T cells, limiting their antitumor efficacy in terms of cytotoxicity and cytokine secretion [9, 10]. The efficacy of checkpoint inhibition, however, extends beyond the simple expression of exhaustion markers, as other immune evasion mechanisms, such as loss of tumor antigen and tumor microenvironment‐induced immunosuppression, also play a significant role [11]. To study patient‐specific responses, immunocompetent *ex vivo* tissue models are a powerful tool [12]. The cultivation of *ex vivo* human lung slices is well established [13, 14, 15, 16]. However, e*x vivo* cultivation of human tumor lung tissue is not yet as widely utilized. Two studies have been published, which mainly utilized histology or imaging to analyze the susceptibility to immune‐checkpoint inhibition in *ex vivo* human tumor lung tissue slices [17, 18]. Aspects such as the immune characterization of different lung regions, tissue cytokine secretion, and specific immune cell responses, however, remain understudied in the literature. Thus, a clearer understanding of patient‐derived tissue composition is needed to reveal how the tumor microenvironment shapes immune responses.

In the present study, precision‐cut tissue slices derived from non‐small‐cell lung cancer (NSCLC), adenocarcinoma, and lung metastasis samples have been used to investigate the native tissue microenvironment and allow for the in‐depth characterization of immune cell responses in defined areas of the tumor and surrounding tissue. This approach could lead to improved treatment strategies in a preclinical setting by analyzing specific activation profiles of T cells in the tumor microenvironment compared with healthy lung tissue from the same patient.

## 2 Results

### 2.1 Tumor‐Border Tissue Slices Are Viable and Highly Enriched with Immune Cells

**FIGURE 1:**
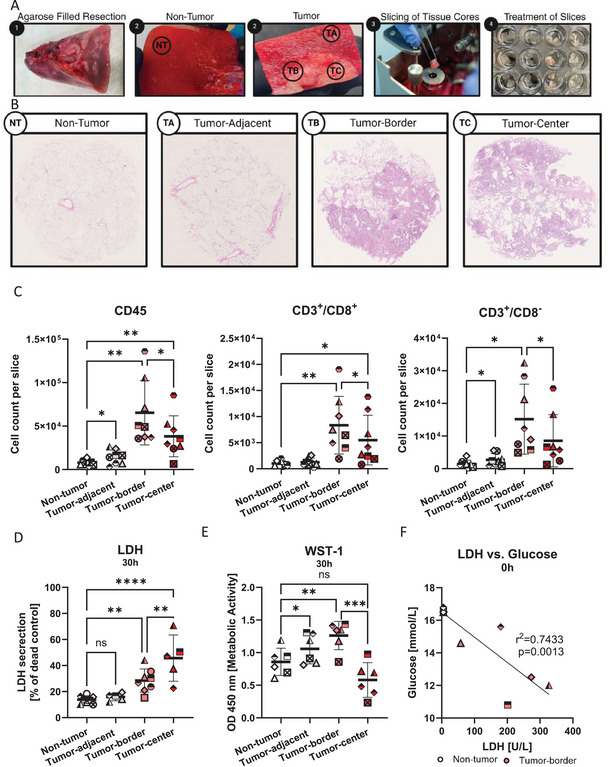
Viability and immune cell frequency of tumor‐derived precision‐cut lung slices are dependent on the allocated lung region. (A) Generation of precision‐cut lung slices derived from non‐tumor, tumor‐adjacent, tumor‐border, and tumor‐central lung tissue. (B) H&E stains of the four lung regions nearing proximity to the tumor. (C) LDH secretion of the lung regions was normalized to the dead control. (D) Absolute 450 nm OD values of WST‐1 metabolic activity by the lung regions. (E) Correlation between LDH secretion (U/L) and Glucose content (nmol/L) in the supernatant in non‐tumor versus tumor‐border tissue. (F) Total cell counts per PCLS slice of CD45^+^, CD8^+^, and CD8^‐^T cells. Each shape represents one donor. *n* = 5–8, RM one‐way ANOVA + SD, Linear regression, *p < 0.05, **p < 0.01, ***p < 0.001.

Varied responses to immunotherapies are, in part, a result of heterogeneous, patient‐specific tumor microenvironments. To investigate individual immune responses *ex vivo*, we first examined the cell composition and viability of precision‐cut tissue sections prepared from different locations within and around the lung tumor (non‐tumor, tumor‐adjacent, tumor‐border, and tumor‐central). Tissue classification was based on macroscopic and histological analysis (Figure 1A,B). We detected no tumor cells in both the non‐tumor and tumor‐adjacent regions. Tumor‐border slices consisted of part tumor tissue and part adjacent lung tissue (Figure 1A), while tumor‐central tissue was rich in tumor cells, with the remaining stroma intermixed with immune cells, as determined by histology (Figures S3 and S4). Similarly, flow cytometry revealed significantly fewer EpCAM^high^/CD45^−^ tumor cells in non‐tumor compared with tumor‐border (21%) and tumor‐central (40%) tissue (Figure S5A). Furthermore, the immune cell counts in tumor‐border slices were significantly higher compared with non‐tumor tissue (CD45: 8.3‐fold; CD3^+^/CD8^+^: sixfold; and CD3^+^/CD8^−^: sevenfold) as well as tumor‐central tissue (Figure 1C). After 30 h of cultivation, both non‐tumor and tumor‐adjacent tissue were viable as confirmed by LDH and WST‐1 assays, and as previously described with non‐tumor lung slices [14]. Comparison of viable cell frequency and CD8/PanCK‐stained IHC‐stained samples before and after cultivation also revealed no changes in viability or tissue structure after cultivation (Figure S6A,B). Interestingly, the tumor‐border released higher levels of LDH (2.7‐fold) and had higher WST‐1 activity (1.56‐fold) compared with non‐tumor slices (Figure 1D,E), suggesting a higher prevalence of metabolically active cells. Indeed, flow cytometry confirmed that the tumor‐ border had the highest live cell percentages and counts (7.5 × 10^5^/slice) compared with the other regions (Figure S5B,C). In contrast to the other lung regions, tumor‐central slices showed reduced viability upon cultivation (Figure 1D). Finally, the tumor‐border displayed tumor characteristic parameters, evidenced by a correlation between high LDH release and high glucose uptake, as measured in the supernatant (Figure 1F). Taken together, tumor‐border slices displayed the highest metabolic activity, the highest live cell counts, and contained the highest total counts of immune cells as well as T cells. Thus, the tumor‐border region is well‐suited for further investigation of immune responses in the tumor microenvironment.

### 2.2 Tumor‐Border Slices Reflect the Lung Tumor Microenvironment After Cultivation

**FIGURE 2:**
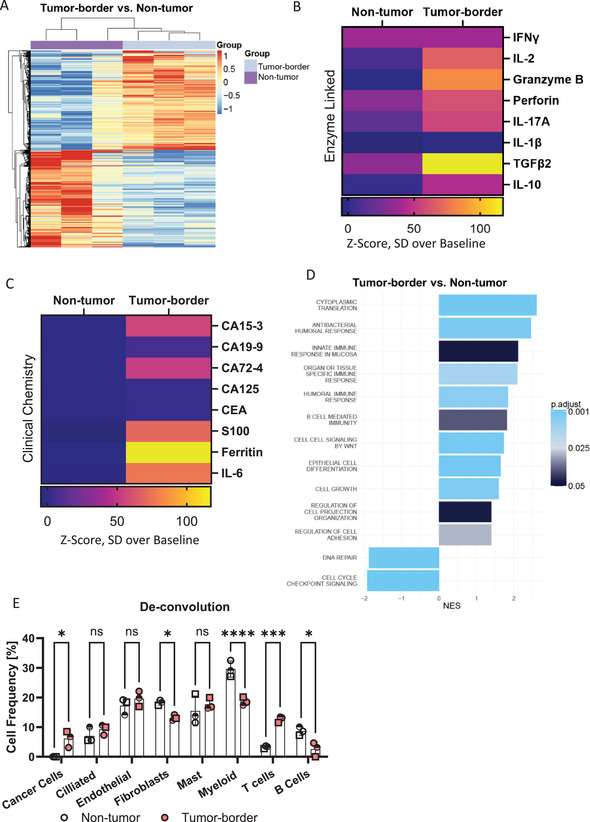
Following 30 h of cultivation, patient‐derived tumor slices maintain a tumor microenvironment that mirrors the characteristics of lung cancer. (A) Expression heatmap of differentially expressed genes (adjusted *p*‐value <0.05) between tumor‐border (purple) and non‐tumor (light blue) (red = upregulated; blue = downregulated compared with the average expression in all samples) (three donors). (B) Clinical chemistry parameters measured in the supernatant of lung slices (Z‐score normalized) (five donors). (C) Supernatant cytokine measurements (Z‐score normalized) of paired non‐tumor and tumor‐border slices (14 donors). (D) Barplot of enriched Gene Ontology (GO) Biological Process (BP) pathways for the comparison between tumor‐border and non‐tumor groups. The *x*‐axis represents the normalized enrichment score (NES), indicating the degree of enrichment for each pathway, with positive values indicating overrepresentation and negative values indicating underrepresentation; the color indicates the adjusted *p*‐value. (E) Deconvolution of bulk RNA‐seq data. All data were collected 30 h postculture. *n* = 3–5, ratio paired *t*‐test, *p < 0.05, **p < 0.01, ***p < 0.001, ****p < 0.0001.

**FIGURE 3:**
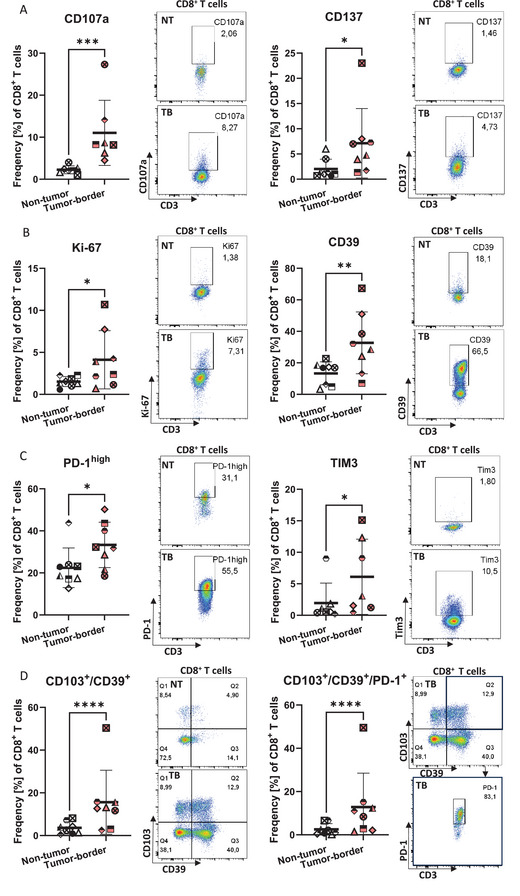
CD8^+^ T cells at the tumor‐border highly express early and late activation markers. Expression of (A) CD137, CD107a (B) Ki‐67, CD39, (C) PD‐1 and TIM3, (D) CD103/CD39 and CD103/CD39/PD‐1 on CD8**^+^** T cells in non‐tumor and tumor‐border slices. Including representative flow cytometry plots. Each donor is represented by one shape. *n* = 6–8, ratio‐paired *t*‐test + SD, *p < 0.05, **p < 0.01, ***p < 0.001, ****p < 0.0001.

To study the effect of the tumor microenvironment on T cell immune responses, the immune status of the tissue is highly relevant. Thus, we analyzed the donor‐matched non‐tumor and tumor‐border slices after cultivation using RNA‐sequencing (RNA‐seq) from three NSCLC adenocarcinoma samples. The expression profiles of the three analyzed donors show good concordance. After 30 h of cultivation, over 4932 differentially expressed genes (DEGs) were identified (2439 upregulated and 2493 downregulated) (Figure 2A; Figure S7). The DEGs included genes involved in cell proliferation and migration (e.g., COL11A1), tumor‐promoting factors that degrade the extracellular matrix (e.g., MMP11), as well as immunosuppressive (TGFB1) and proinflammatory responses (TNFRSF6B, IFNGR2, GSDMB) (Table S2) (Figure 2A) [19, 20, 21, 22]. In line with this, analyses of proteins in the supernatant of the tissue slices from 14 donors showed that anti‐inflammatory cytokines like TGFβ2 and IL‐10, as well as proinflammatory cytokines like IFNγ, IL‐2, Granzyme B, Perforin, IL‐17A, and IL‐1β were highly secreted by tumor‐border compared with non‐tumor slices (Figure 2B; Tables S3 and S4). Additionally, clinical chemistry data of secreted proteins showed that tumor‐border slices, but not non‐tumor slices, produced known tumor‐associated markers, including CA15‐3, CA19‐9, CA72‐4, CA125, CEA, S100, ferritin, and IL‐6 (Figures 2C; Tables S3 and S4) [23, 24, 25, 26]. In support of these classical tumor microenvironment characteristics, gene ontology (GO) enrichment analysis of the DEGs revealed upregulated pathways involved in cell growth, mucosal, and tissue‐specific immune responses in tumor‐border slices (Figure 2D). Further, deconvolution of the RNA‐seq data revealed that, after cultivation, relevant cell types, including endothelial cells, fibroblasts, mast cells, myeloid cells and B cells, were preserved in the tissue of all three donors. Furthermore, the deconvolution supports the flow cytometry findings in Figure 1C, in that the frequency of T cells in tumor‐border tissue slices was significantly higher than in non‐tumor tissue (∼13% vs. 3%) (Figure 2E). The above‐mentioned T cell‐associated cytokines were accompanied by a higher frequency of activated cytotoxic CD107a^+^ (2% vs. 11%) and CD137^+^ (3% vs. 6%) CD8^+^ T cells in tumor‐border lung tissue slices compared with non‐tumor slices (Figure 3A). Additionally, six out of eight donors exhibited a higher frequency of the proliferation marker, Ki‐67 expression in CD8^+^ T cells of tumor‐border lung tissue slices (1.5% vs. 4.1%) (Figure 3B). Markers of T cell exhaustion, CD39, PD‐1, and TIM3 were significantly higher expressed in the tumor‐border compared with non‐tumor lung tissue slices (Figure 3B,C). Additionally, CD103 and PD‐1 are expressed on CD39^+^/CD8^+^ T cells more so at the tumor‐border compared with non‐tumor tissue, indicating their tissue residency and antigen experience (Figure 3D). Taken together, these results emphasize that tumor‐border tissue shows distinct immune activation and inflammation profiles compared with non‐tumor tissue, with cytotoxic activated T‐cell phenotypes bearing markers of T cell exhaustion.

### 2.3 Antigen‐Independent T Cell Activation in the Tumor‐Border Induces Tissue Cytokine Responses

**FIGURE 4:**
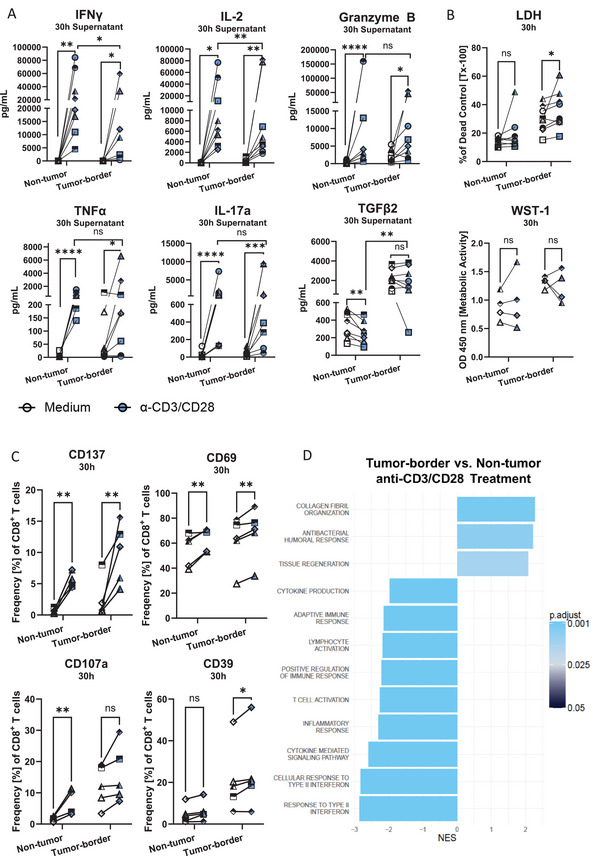
T cells in tumor‐border lung slices respond to anti‐CD3/CD28 and induce proinflammatory responses in the tissue. (A) Multiplex cytokine analysis of IFNγ, IL‐2, Granzyme B, TNF‐α, IL‐17a, and TGFβ (*n* = 8). (B) LDH release normalized to dead control and WST‐1 assay (*n* = 4 or 5); (C) flow cytometry of expression of CD137, CD69, CD107a, and CD39 on CD8^+^ T cells (*n* = 5) and (D) Barplot of enriched Gene Ontology (GO) Biological Process (BP) pathways for the comparison between tumor‐border and non‐tumor groups after anti‐CD3/CD28 treatment. The *x*‐axis represents the normalized enrichment score (NES), indicating the degree of enrichment for each pathway, with positive values indicating overrepresentation and negative values indicating underrepresentation; the color indicates the adjusted *p*‐value (*n* = 3). Paired *t*‐tests, *p < 0.05, **p < 0.01, ***p < 0.001.

As demonstrated above, tumor‐border tissue displays a clear immune activation status. To examine if the described T cell phenotypes exhibit functionality and can thus induce an immune response, we treated the tissue slices with antigen‐independent stimulation using anti‐CD3/CD28 for 30 h. Proinflammatory cytokines associated with T cell responses were significantly increased by anti‐CD3/CD28 in both tissue types after stimulation, as evidenced by a significant increase in IFNγ (non‐tumor: 1,368‐fold; tumor‐border:149‐fold), IL‐2 (non‐tumor: 432‐fold; tumor‐border: 78‐fold), Granzyme B (non‐tumor: 180‐fold; tumor‐border: 7.6‐fold), TNFα (non‐tumor: 105‐fold; tumor‐border: 7.9‐fold), and IL‐17A (non‐tumor: 62‐fold; tumor‐border: 57‐fold). Contrarily, the anti‐inflammatory cytokine TGFβ was only significantly downregulated in non‐tumor slices (0.7‐fold). Notably, the response of non‐tumor tissue was significantly stronger when comparing final concentrations of IFNγ and IL‐2 after treatment (Figure 4A). This difference in response magnitude is also evident in the lower‐fold changes seen for all other cytokines secreted by the tumor‐border compared with non‐tumor tissue. Interestingly, cytokine secretion did not correlate with immune cell count (Figure S8A,B), nor was there a clear trend when considering baseline IFNγ, TGFβ secretion without treatment, CD8^+^ T cell count, or PD‐1 expression (Table S5). Furthermore, stimulation with anti‐CD3/CD28 increased LDH release in tumor‐border slices (1.4‐fold compared with the medium control) (Figure 4B). In concurrence with the secretion of inflammatory cytokines, T cells in both tissue types significantly upregulated the expression of activation markers CD137 and CD69. The activation and exhaustion marker CD39 was only significantly upregulated in tumor‐border slices (mean: 21.3% vs. 24.5%), while the degranulation marker CD107a was only upregulated on CD8^+^ T cells in non‐tumor tissue slices (mean: 1.8% vs. 7.9%) (Figure 4C). Interestingly, pathway analysis from bulk RNA‐seq of three donors revealed that, despite the induction of an inflammatory cytokine response, inflammatory pathways such as T cell proliferation and the adaptive immune response were significantly downregulated in tumor‐border compared with non‐tumor lung tissue slices (Figure 4D). This RNA‐seq data support the observed lower fold changes of cytokine secretion by tumor‐border slices. Taken together, protein and RNA‐seq data suggest that tumor‐border resident immune cells have limited capacity to respond to immune activation but can be activated using antigen‐independent stimuli with varying magnitudes of whole‐tissue response.

### 2.4 Nivolumab Induces Proinflammatory Responses in Tumor‐Border but Not Non‐tumor Tissue

**FIGURE 5:**
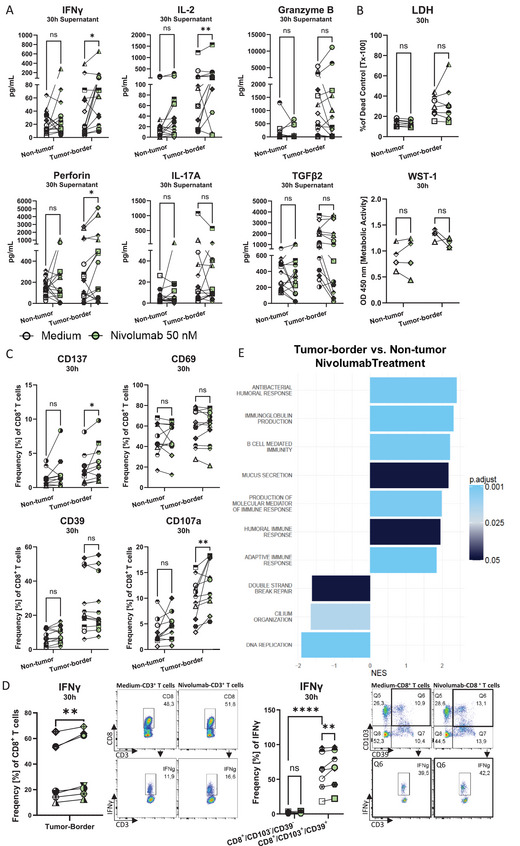
Nivolumab treatment induces proinflammatory pathways in tumor‐border but not non‐tumor slices. (A) Multiplex cytokine analysis of IFNγ, IL‐2, Granzyme B, Perforin, IL‐17a, and TGFβ (*n* = 14). (B) LDH release normalized to dead control and WST‐1 assay (*n* = 4 or 5). (C) Flow cytometry of expression of CD137, CD69, CD107a, and CD39 on CD8^+^ T cells (*n* = 11). (D) Intracellular IFN‐γ in CD8**^+^** T cells and CD8^+^/CD103^+^/CD39^+^ vs. CD8^+^/CD103^−^/CD39^−^ T cells after nivolumab treatment measured by flow cytometry (*n* = 5 or 7). (E) Barplot of enriched Gene Ontology (GO) Biological Process (BP) pathways for the comparison between border and non‐tumor groups after Nivolumab treatment. The *x*‐axis represents the normalized enrichment score (NES), indicating the degree of enrichment for each pathway, with positive values indicating overrepresentation and negative values indicating underrepresentation; the color indicates the adjusted *p*‐value (*n* = 3). Paired *t*‐tests, *p < 0.05, **p < 0.01.

Anti‐PD‐1 treatment in the clinics is effective in some cancer patients with high expression of PD‐L1 [27]. PD‐L1 alone, however, is not sufficient to predict outcome, as responses still vary depending on the T cell functionality [28]. To reflect these varied responses *ex vivo*, we investigated CD8^+^ T cell activity and cytokine secretion of lung tissue slices upon anti‐PD‐1 treatment using Nivolumab. Notably, after treatment, proinflammatory cytokine secretion was significantly increased by tumor‐border, but not by non‐tumor lung tissue slices, compared with the medium control, evidenced by higher levels of IFNγ (1.7‐fold), IL‐2 (1.4‐fold), and Perforin (1.5‐fold) (Figure 5A). Though the effect on these cytokines was significant, not all donors showed a response in all cytokines, indicating a donor‐dependent response to the treatment. Other cytokines relevant to tumor clearance, such as Granzyme B and IL‐17, were also regulated in a patient‐specific manner, with increased secretion of both mediators observed in six out of eight and four out of eight donors, respectively. Similarly, decreased TGFβ secretion was observed in six out of eight donors, indicating reduced anti‐inflammatory processes. These responses, however, did not correlate with the proximity of CD8^+^ T cells to tumor cells (Figure S8A,C,D). While LDH release and cytokine secretion were donor‐dependently induced (Figure 5A,B), CD8^+^ T cells were activated in tumor‐border slices of most donors as denoted by a significant upregulation of CD137 (mean: 2.7% to 3.7%), CD107a (mean: 9.8% to 12.9%) and intracellular IFNγ (mean: 33.1% to 38.4%) expression (Figure 5C,D). Interestingly, CD8^+^/CD103^+^/CD39^+^ T cells expressed high levels of IFNγ, which was further upregulated upon Nivolumab treatment in comparison to CD8^+^/CD103^−^/CD39^−^ T cells (Figure 5E). GO‐enriched pathway analysis of Nivolumab‐treated slices revealed upregulated pathways related to immunoglobulin production, mucosal immune response, and humoral immune response in tumor‐border lung tissue slices compared with non‐tumor slices (Figure 5D). Taken together, the data indicate specific responses to anti‐PD‐1 treatment in the tumor‐border only, while non‐tumor tissue did not show increased immune activation. While there was a significant increase in inflammatory responses and T cell activation in tumor‐border tissue, these responses were varied and strongly donor‐dependent.

## 3 Discussion

Efficacy of immunotherapies is critically dependent on the individual immune cell composition of each patient's specific tumor microenvironment. Previous publications have demonstrated that, in the context of NSCLC, a high infiltration of T cells with a proinflammatory phenotype, expressing molecular breaks such as PD‐1, is positively correlated with a response to immune checkpoint inhibition [29, 30]. Expression of exhaustion markers alone, however, is not sufficient to predict outcome [31]. The current paper demonstrates the variability in the magnitude of immune responses after T cell stimulation in a spatial and patient‐specific context of the immune microenvironment. In other studies, tumor‐infiltrating lymphocytes were isolated and cultivated *ex vivo*, with and without autologous tumor, to analyze T cell clones and study their antitumor reactivity [6]. Although this method can predict the reactivity of tumor‐reactive T cells, these studies lack the context of an immunosuppressive tumor microenvironment. To account for this, *ex vivo* patient‐derived tumor tissue fragments and tumor lung slices were used to study response to immunotherapies [17, 32]. Here, the responses were mainly studied using histology and imaging, and only investigated the tumor tissue itself. Furthermore, because cultivation of tissue slices inevitably induces changes in the tissue [33, 34], we have stringently assessed the tissue viability and established a short cultivation duration to better study early immune responses. In the present study, we combine both phenotypic and functional analyses of tissue‐residing T cells within the lung tissue microenvironment of defined regions of the lung (non‐tumor, tumor‐adjacent, tumor‐border, and tumor‐center). In utilizing precision‐cut lung slices of defined regions, we could better standardize the size and location of the area of interest.

In lung adenocarcinoma, inflammation in the tumor‐adjacent lung is a predictor of clinical outcome [35]. Additionally, spatial immunophenotyping of NSCLC samples revealed that the proximity of immune cells to tumor cells has a prognostic impact [36, 37]. Our study supports these findings, as the inflammatory responses were dependent on the location and density of CD8^+^ T cells. In the same study, however, the proximity of Tregs to effector CD8^+^ T cells negated the positive impact of high CD8^+^ T cell densities [36]. Similarly, we did not identify a correlation between cytokine response and the distance between CD8^+^ T cells and PanCK‐expressing cells, which may be explained by high TGFβ levels, indicative of the presence of immunosuppressive cells in the microenvironment.

NSCLC and various metastases have been extensively immunophenotyped [38, 39]. Our current data complement the existing data, as the tumor‐border slices display DEG and secrete pro‐ and anti‐inflammatory factors associated with a classical tumor microenvironment. Notably, we have demonstrated that the tumor‐border is well‐suited for investigating patient‐specific responses to checkpoint inhibitors in the context of all cells within the tissue slice. Interestingly, the functional status of T cells in the tumor‐border of NSCLC was correlated with cancer recurrence [37]. The distinct phenotype of PD‐1^high^, TIM3, CD39‐expressing CD8^+^ T cells within the tumor‐border is in line with published works addressing T cell phenotypes in NSCLC samples [40]. Tissue‐resident CD8^+^/CD103^+^/CD39^+^ T cells are suggested to be tumor‐specific in other contexts, which we support in finding that these secrete IFN‐γ upon stimulation with Nivolumab [6, 7]. Although the phenotype of these lung‐resident T cells is well defined, the activation profiles within the lung tumor microenvironment have not yet been studied. Our current data provide information on the dynamic effect of T cell stimuli within the tumor microenvironment during an early time point. Although the tissue in this study originated from NSCLC samples and lung metastasis samples, the cytokine and T cell responses could not be stratified according to the tumor of origin based on these factors alone.

Overall, stimulation of T cells within the tissue led to donor‐dependent differences resulting in a variable magnitude of cytokine secretion and overall response in the tumor‐border and non‐tumor tissue. For example, the secretion of IFNγ at the tumor‐border across all donors after treatment with anti‐CD3/CD28 ranged from 440 to 60,000 pg/mL. Lower response rate after treatment with Nivolumab compared with anti‐CD3/CD28 is because anti‐CD3/CD28 activates all T cells in the tissue, while Nivolumab only targets PD‐1‐expressing, antigen‐experienced T cells [41], resulting in a weaker tissue response. These results highlight the significant variability in immune responses, not only between different donors but also depending on the specificity of the stimuli. Taken together, we have demonstrated that activation of CD8^+^ T cells within the native lung and tumor lung microenvironment induces an inflammatory response of the tissue *ex vivo*. In a spatially defined and functional manner, we have also demonstrated that T cells in both non‐tumor as well as tumor‐border tissue respond to polyclonal activation via anti‐CD3/CD28. In contrast, treatment with Nivolumab induces an inflammatory response primarily at the tumor‐border and may primarily activate tumor‐specific CD8^+^ T cells. Furthermore, both stimuli demonstrated donor‐dependent differences, as the magnitude of responses was varied.

In this study, we present a platform for assessing patient‐specific immune responses within the human tissue microenvironment for preclinical applications. The short cultivation period enables detection of early immune activity, as measured by flow cytometry and cytokine analysis, within five days of tissue collection. Integrating *ex vivo* tissue slices into clinical studies and linking early responses to treatment outcomes could help predict patient response, which will be subject to future studies. With automated tissue slicing, this model holds potential for clinical use before therapy initiation. Taken together, studying these response profiles may help to improve the efficacy of newly developed immunotherapies and better evaluate immune responses to checkpoint therapies in the clinic.

### 3.1 Data Limitations and Perspectives

This study demonstrates the use of immunocompetent *ex vivo* tumor lung slices in a preclinical setting, in particular, analyzing CD8^+^ T cell responses in the tumor microenvironment. However, a limitation of these *ex vivo* slices is that to ensure adequate viability, a culture duration of a maximum of 30 h can be reached. Additionally, this model is suitable for analyzing local immune responses, as no immune cell infiltration takes place. Further perspectives include optimizing culture duration using microfluidic chip systems to analyze later time points of immunomodulation. Finally, in future experiments, spatial transcriptomics would be valuable to analyze immune‐tumor‐stroma interactions in a spatial context.

## 4 Materials and Methods

### 4.1 Ethics Statement and Patient Samples

Experiments with patient‐derived tissue slices were performed according to the Code of Ethics of the World Medical Association and Declaration of Helsinki and approved by the local ethics committee of the Hannover Medical School (approval numbers 2923‐2015 and 3342‐2016), including written informed consent to use human lung material from all donors. Patient samples consisted of 13 NSCLC adenocarcinoma patients, one carcinoid lung tumor patient, and four patients with metastasis from colorectal, uterus, renal cell, and salivary gland carcinomas to the lung (Table S1).

### 4.2 Generation and Cultivation of Precision‐Cut Lung and Tumor Lung Slices

Lung tumor resections were filled with 4% low‐melting agarose mixed 1:1 with DMEM/F12. The filled lung was left to polymerize on ice and cut into slabs. The regions of interest were punched out as 8 mm cores. Macroscopically unaffected tissue was cut into 300 µm slices, while tumor‐affected slices were cut into 275 µm slices. The thinner slices are to account for the denser tumor tissue to improve viability. Non‐tumor slices were generated from tissue at least 10 cm away from the tumor, while tumor‐adjacent tissue was within 3 cm away from the tumor center (Figure 1A). Tumor‐border slices were macroscopically determined to be part tumor tissue, while tumor‐central slices were derived from visually nonnecrotic but restructured tissue near the center of the tumor. Processing of the lung and generation of slices were conducted as previously published [14]. Two slices were cultivated in 500 µL DMEM/F12 + P/S (Thermo Fisher, 11039021 and 15070063). Treatment with anti‐CD3/CD28 Dynabeads (Thermo Fisher, 11131D) was conducted by adding 5 µL of beads. Nivolumab (Selleckchem, A2002) was added at 50 nM to the culture medium. Slices were cultivated till the latest possible time point (30 h) to measure immune responses, while ensuring adequate tissue viability.

### 4.3 Tissue Digestion and Flow Cytometry

After culture, six lung slices were pooled, minced into small pieces, and covered with 1 mL dissociation mix comprised of 1 mg/mL Collagenase D (Merck, 11088858001) and 0.055 mg/mL DNase I (Merck, 4536282001) and left to shake at 200 rpm at 37°C for 1 h. The slices were then mechanically dissociated using a pipette, vortexed, and filtered through a 100 µm strainer. The single‐cell suspension was centrifuged at 350×*g* for 10 min. The pellet was resuspended in 300 µL FACS buffer (PBS + 1% FCS + 2 µM EDTA) with 1:100 Tru‐Stain FcX (Biolegend, 422302) and blocked for 10 min on ice. Subsequently, the suspension was split into three equal parts to stain three panels. Panels 1, 2, 3, and 4 had the same base markers: CD45‐PerCP (Biolegend 304026), CD3‐AF700 (Biolegend 300424), CD8‐BV510 (Biolegend 301048), CD103‐PE (Biolegend 350206), CD39‐BV421 (Biolegend 328214), and Zombie NIR. (Biolegend 423106) Panel 1 included the additional markers CD49a‐FITC (Biolegend 328308), CD137‐PE‐Cy7 (Biolegend 309817), CD69‐BV650 (Biolegend 310934), and Ki67‐APC (Biolegend 350514). Panel 2 included CD73‐FITC (Biolegend 344016), Tim3‐BV650 (Biolegend 34502), PD‐1‐PE‐Cy7 (Biolegend 329918), and TIGIT‐APC (Biolegend 372706). Panel 3 included EpCAM‐PE‐Cy7 (Biolegend 324222), E‐cadherin‐APC (Biolegend 324108), HLA‐DR‐BV650 (Biolegend 307650), and CD107a‐FITC (Biolegend 328606). Panel 4 included the additional markers IFNγ‐FITC (Biolegend 502515), Granzyme B‐APC (Biolegend 515405), CD69‐BV650 (Biolegend 310934), and CD137‐PE‐Cy7 (Biolegend 309817). Surface stain markers were stained for 30 min at 4°C. Intracellular staining was conducted according to the manufacturer's protocol using the FoxP3 Fix Perm Kit, where cells were fixed for 20 min (Thermo Fisher, 00552300). The gating strategies were used for Figures 1, 3, 4, and 5 (Figures S1 and S2).

### 4.4 Histology

At given time points (0 h or 30 h after cultivation), lung slices were fixed in 2% PFA overnight. Subsequently, the slices were washed with PBS and stored in 0.05% sodium azide until paraffin embedding, slicing, and hematoxylin and eosin (H&E) staining, and mounted for light microscopy. Additional unstained slides were used for immunohistochemistry.

### 4.5 Immunhistochemistry and Image Analysis

For duo‐plex staining, the slides were initially treated for heat‐antigen retrieval at a pH level of 8. Slides were first stained for CD8 using a primary mouse monoclonal anti‐CD8 antibody (Abcam ab17147), followed by a secondary antibody (Dako REAL Link, biotinylated secondary antibody (AB2)) and 3,3′‐diaminobenzidine (DAB) as chromogen. Secondly, staining against cytokeratin (CK) was performed using a mouse monoclonal anti‐pan‐cytokeratin antibody (Sigma‐Aldrich C2562), followed by a secondary antibody (goat‐anti‐mouse Jackson ImmunoResearch 115‐065‐155) and DAKO REAL chromogen red (K500511‐2). For counterstaining, hematoxylin was used. Stained slides were digitized using the Hamamatsu S‐210 slide scanner (Hamamatsu Photonics Europe GmbH, Herrsching am Ammersee, Germany). Image analysis was performed with the Visiopharm Image analysis software (Visiopharm A/S, Hørsholm, Denmark) using the nuclei detection AI algorithm to detect the cells and further classify the cells into CD8‐positive, CK‐positive, and negative cells. Finally, CD8‐positive cells with a distance of 3 µm or less to CK‐positive cells were identified.

### 4.6 Viability Assays

The viability of lung slices was determined either by lactate dehydrogenase (LDH) release assay from supernatants (LDH Cytotoxicity Assay Kit, Roche Diagnostics) or from the tissue mitochondrial metabolic activity with Cell Proliferation Reagent WST‐1 (Roche Diagnostics) according to the manufacturer's instructions. Triton X‐100 (1% in PBS (Sigma))‐treated lung slices were utilized as a reference (dead) control. For LDH measurements, 1:10 dilutions of tumor slice samples and Triton X‐100 controls were applied to adjust for high optical density values. Absorbance was measured at 490 and 630 nm as reference wavelengths for LHD, and 420–480 nm with reference to 690 nm for WST‐1 using a microplate reader (Microplate Reader Infinite 200 Pro Tecan Group, Männedorf, Switzerland).

### 4.7 Clinical Chemistry Parameters

Measurements from tissue supernatants were performed using Roche Cobas 8000 modular analyzer series systems with modules e801 and c702 according to the manufacturer's specifications. Measurements included LDH, Glucose, CA15‐3, CA19‐9, CA72‐4, CA125, CEA, S100, Ferritin, and IL‐6.

### 4.8 Multiplex Cytokine/Enzyme Measurements

Cytokine responses were detected in the supernatant of cultured lung slices using U‐Plex MSD (Mesoscale Discovery) panels. The immuno‐oncology panel entailed: IFNγ, IL‐2, TNFα, IL‐1β, IL‐10, Granzyme A, Granzyme B, IL‐7, IL‐17A, IL‐15 and Perforin. Additionally, TGFβ1, TGFβ2, and TGFβ3 were measured as a separate multiplex. The multiplex assays were measured using the MESO QuickPlex SE 120 MM according to the manufacturer's instructions.

### 4.9 RNA Isolation and Bulk Sequencing

RNA, including miRNA, was isolated using the MagMAX mirVana Total RNA Isolation Kit94 (ThermoFisher Scientific) with modifications as described [42]. RNA quality was checked using an Agilent 5400. Bulk RNA‐sequencing was conducted by Novogene using Illumina sequencing (HWI‐ST1276), PE150 (12G raw data per sample) with poly A enrichment.

### 4.10 Bulk Sequencing Analysis and Deconvolution

Raw bulk RNA‐sequencing (RNA‐seq) data were preprocessed using the nf‐core/rnaseq pipeline v.3.10.1 [43]. Adapter sequences and low‐quality reads were trimmed with TrimGalore v.0.6.7 (https://github.com/FelixKrueger/TrimGalore). Filtered reads were aligned to the Ensembl mouse genome (GRCh38, release 109) using STAR v.2.7.9a [44]. Transcript quantification was performed with Salmon v.1.9.0 [45], using Ensembl gene annotations (release 109). Subsequent analyses were conducted in R v.4.3.1 [46]. Gene‐level counts were generated with tximport v.1.28. Differential expression analysis was performed with DESeq2 v.1.40.2 [47], using the design formula: ∼ Subject.ID + Group + Treatment + Group:Treatment. “Subject.ID” represents donors, “Group” refers to lung slice categories (tumor‐border, non‐tumor), and “Treatment” represents experimental conditions (Medium, anti‐CD3/CD28, Nivolumab). The interaction term Group:Treatment was included to evaluate whether the effect of treatment differed across the lung slice groups. Genes with an adjusted p‐value < 0.05 were considered differentially expressed. Fold change shrinkage was applied using the lfcShrink function (method = “ashr”) [48]. Expression heatmaps were generated from mean‐centered variance‐stabilizing transformed (VST) counts, using heatmap v. 1.0.12. Venn diagrams related to up‐ and downregulated genes across the three donors were generated using the ggVennDiagram package v.1.4.5. Pathway enrichment analysis was performed via Gene Set Enrichment Analysis (GSEA) [49] in clusterProfiler package v. 4.8.3 [50] using GO biological processes from MSigDB (c5.go.bp.v2023.2.Hs) (Liberzon A, Subramanian A, Pinchback R, et al. Molecular signatures database (MSigDB)) [51], with enriched pathways defined by an adjusted *p*‐value < 0.05. GSEA input consisted of HUGO gene symbols, ranked and filtered as previously described [51, 52]. For deconvolution counts per million (CPM), expression values for both non‐tumor and tumor‐border bulk RNA‐seq data were processed using the edgeR package in R. As reference data, a publicly available single‐cell RNA‐seq data [53] set was used. Signature matrices were generated using CIBERSORTx. For this process, the extraction of subsamples comprising 5000 cells from the scRNA‐seq data was performed with Seurat in R. Cell proportions were meticulously maintained as per the original dataset composition. Two distinct signature matrices were formulated: one inclusive of tumor cells and the other exclusive of them. The final step involved the imputation of cell fractions, executed using standard parameters in CIBERSORTx.

### 4.11 Statistics

RM one‐way ANOVA and ratio paired *t*‐test were performed for statistical analysis using GraphPad Prism 10.1.2 (GraphPad, San Diego, CA). Data show mean and standard deviation (SD). Differences were considered statistically significant at *p* < 0.05.

## Author Contributions

Tonia Bargmann, Susann Dehmel, Charline Sommer, and Katherina Sewald wrote the original draft. Tonia Bargmann performed experiments with lung slices. DS performed histology and imaging analysis. Tonia Bargmann, Susann Dehmel, Sebastian Konzok, and Armin Braun conceived and designed the *ex vivo* studies. Tonia Bargmann performed endpoint measurements and data analysis for flow cytometry and cytokine analysis. Bulk RNA‐seq analysis was performed by Renato Liguori, Maximilian Fuchs, and Fulvia Ferrazzi. Clinical Chemistry parameter measurements were conducted by Stephan Halle and Charlotte Schob. Armin Braun and Katherina Sewald provided resources and scientific supervision. Pathological diagnosis and support work with human tissue were performed by Christopher Werlein and Danny Jonigk. Patrick Zardo, Lavinia Neubert, and Hans‐Gerd Fieguth performed the clinical clarification of the patients and removal of the tissue. All authors reviewed and contributed to the final version of the manuscript.

## Conflicts of Interest

The authors declare no conflict of interest.

## Peer Review

The peer review history for this article is available at https://publons.com/publon/10.1002/eji.70060.

## Supporting information




**Supporting Information file 1**: eji70060‐sup‐0001‐SupMat.pdf

## Data Availability

The datasets generated during and/or analyzed during the current study are available from the corresponding author upon reasonable request.
