# Supplementary material for: Activation of CD8⁺ T Cells in the Human Ex Vivo Lung Tumor Microenvironment Using Anti‐CD3/CD28 and Nivolumab
Source: Eur J Immunol. 2025 Sep 15;55(9):e70060. doi: 10.1002/eji.70060 (PMC12435149; doi:10.1002/eji.70060)
Supplement: Supplementary file 1 — Supporting Information file 1: eji70060‐sup‐0001‐SupMat.pdf [file EJI-55-e70060-s001.pdf]

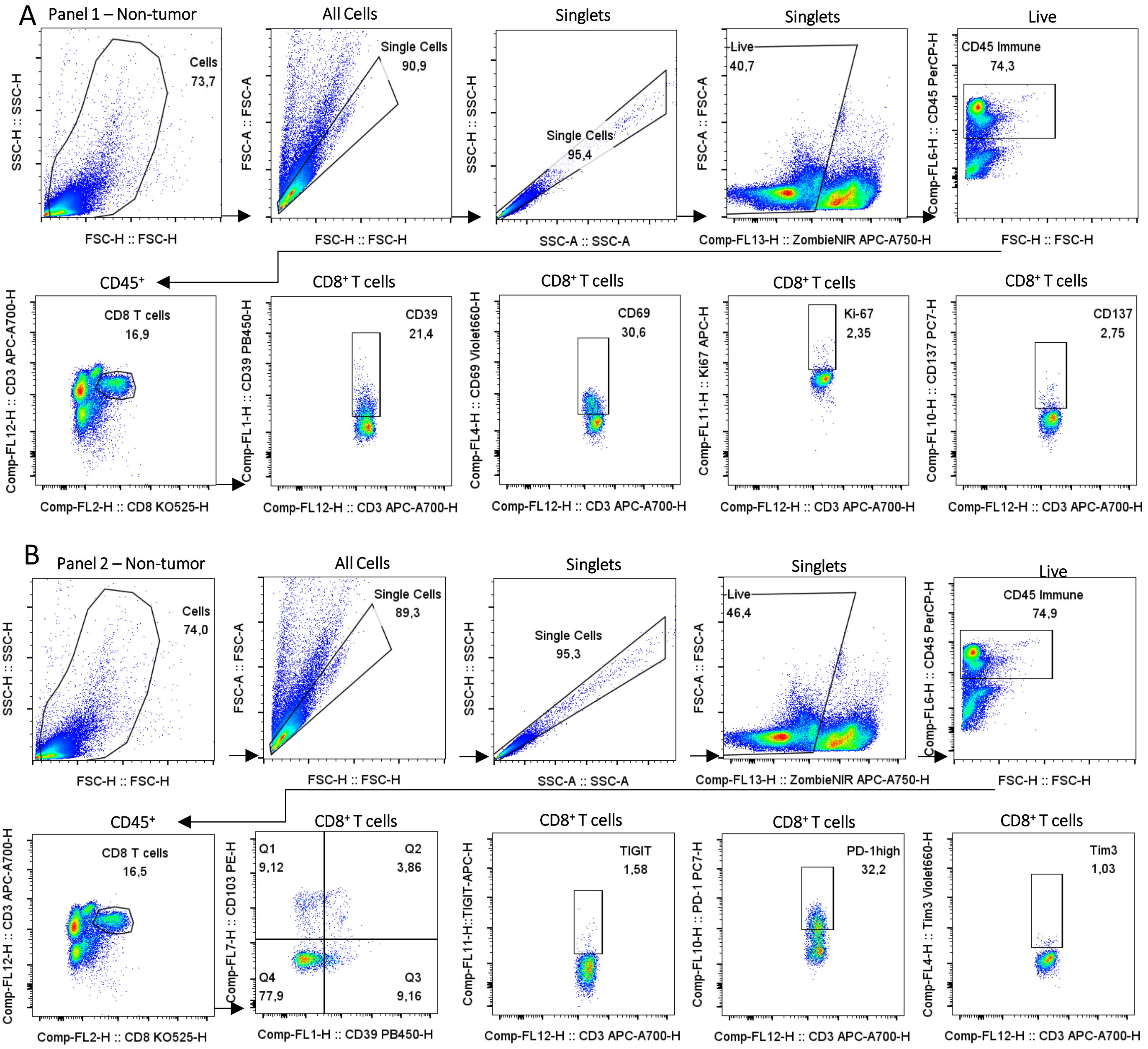

Supplementary Figure 1. *Flow cytometry gating strategy A) Panel 1, fixed intracellular staining B) Panel 2, fixed intracellular staining.*

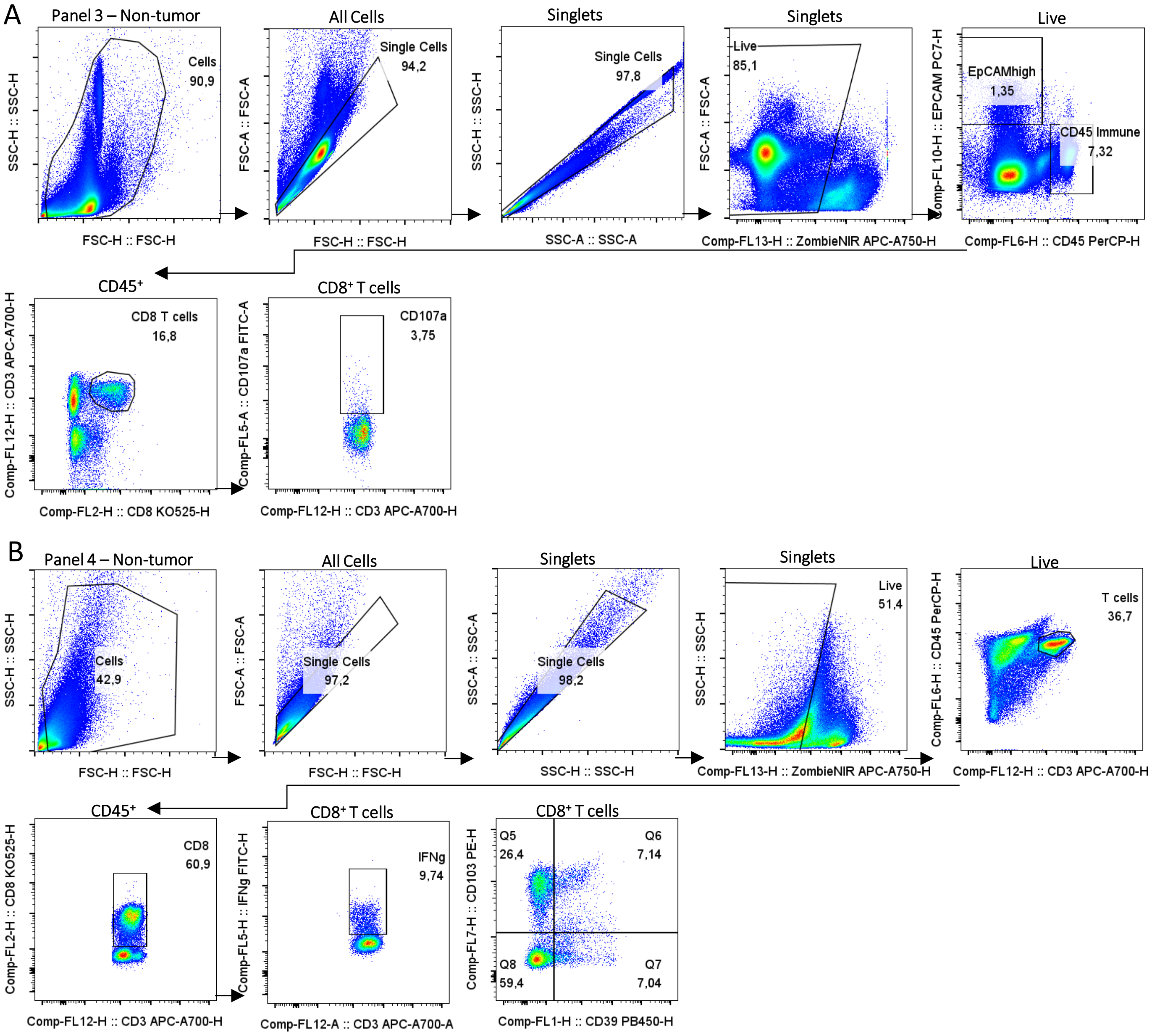

Supplementary Figure 2. *Flow cytometry gating strategy A) Panel 3 B) Panel 4, fixed intracellular staining*

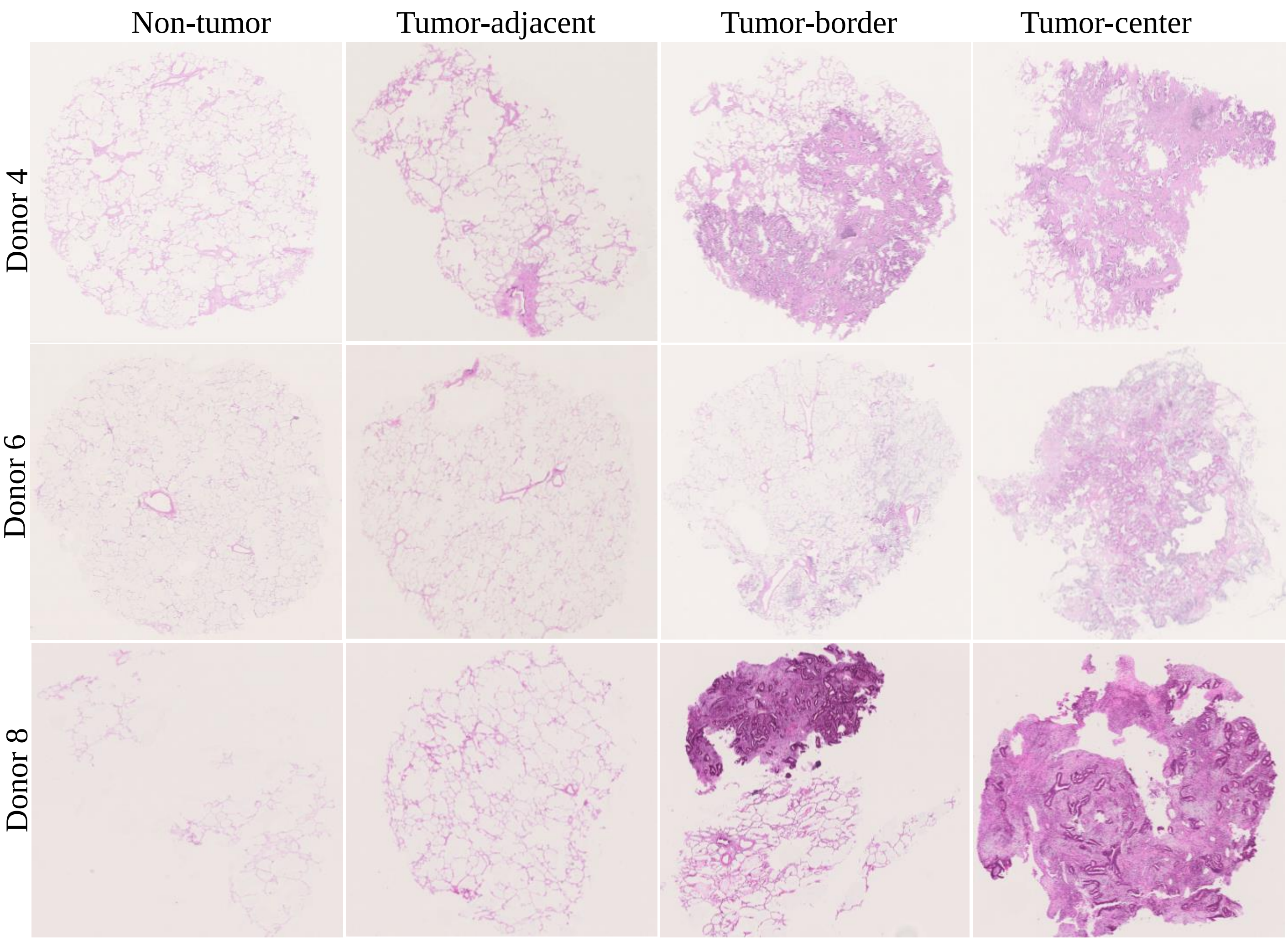

Supplementary Figure 3. No tumor cells are visible in the tumor far slices *A) H&E stains of full lung slices of all four lung regions (Non-tumor, tumor-adjacent, tumor-border and tumor-central) from three representative donors after 30h of culture.*

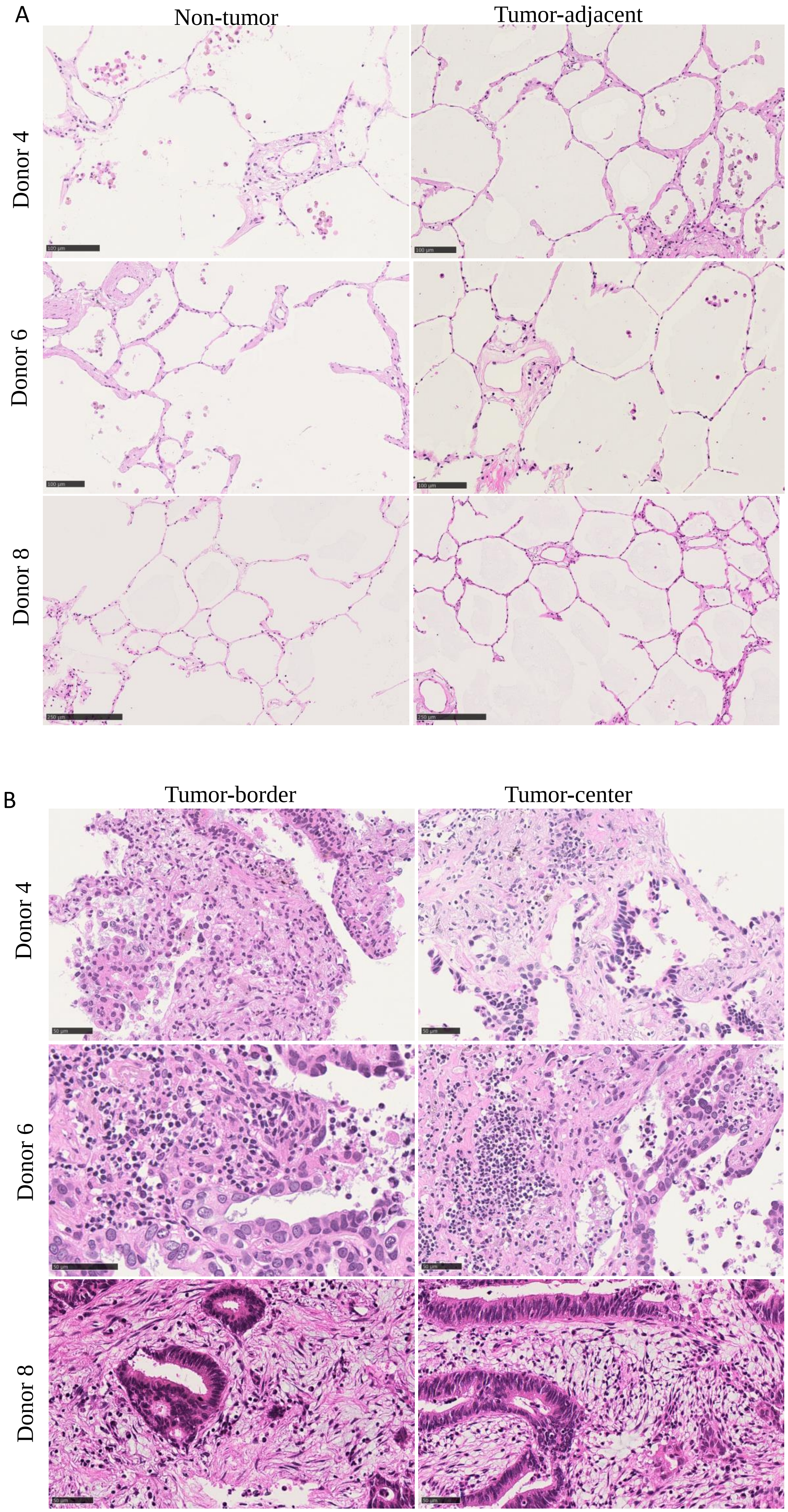

Supplementary Figure 4 .H&E stains of lung slices from different regions *A) H&E stains of select area from non-tumor and tumor-adjacent and. B) tumor-border and tumor-central slices after 30h of culture.*

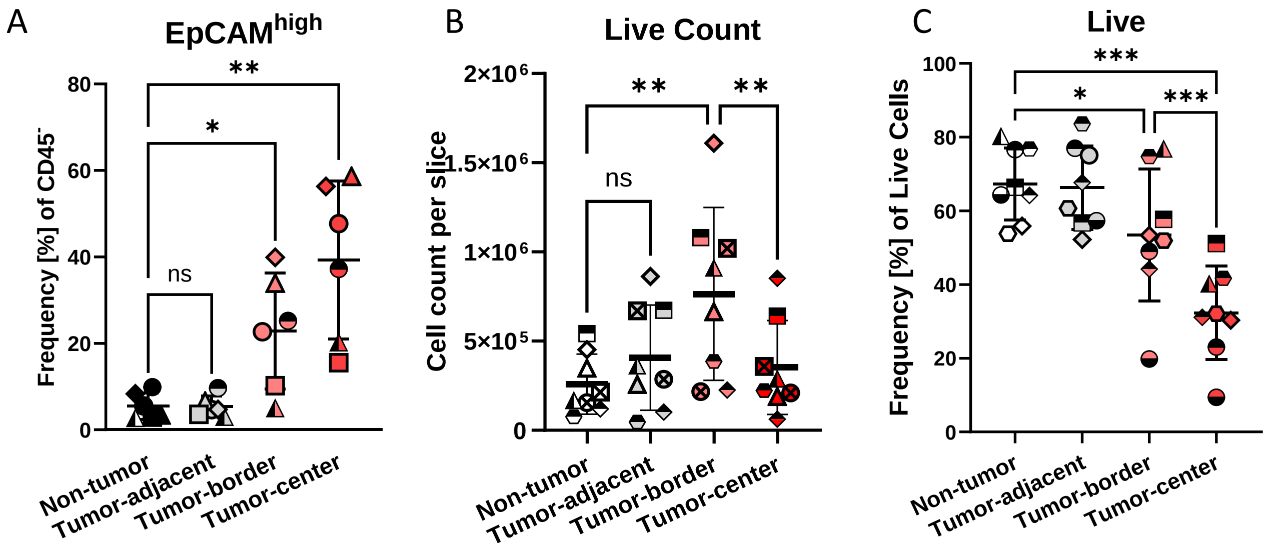

Supplementary Figure 5. Precision cut tissue slices approaching tumor proximity display varying frequency of tumor cells, live cell count and live cell frequency. *A) Cell frequencies of EPCAM^{high} cells B) Cell counts and C) cell frequencies of live cells in four lung regions as mesured by flow cytometry. n=6-8, RM one-way ANOVA, *p<0.05, **p<0.01, ***p<0.001.*

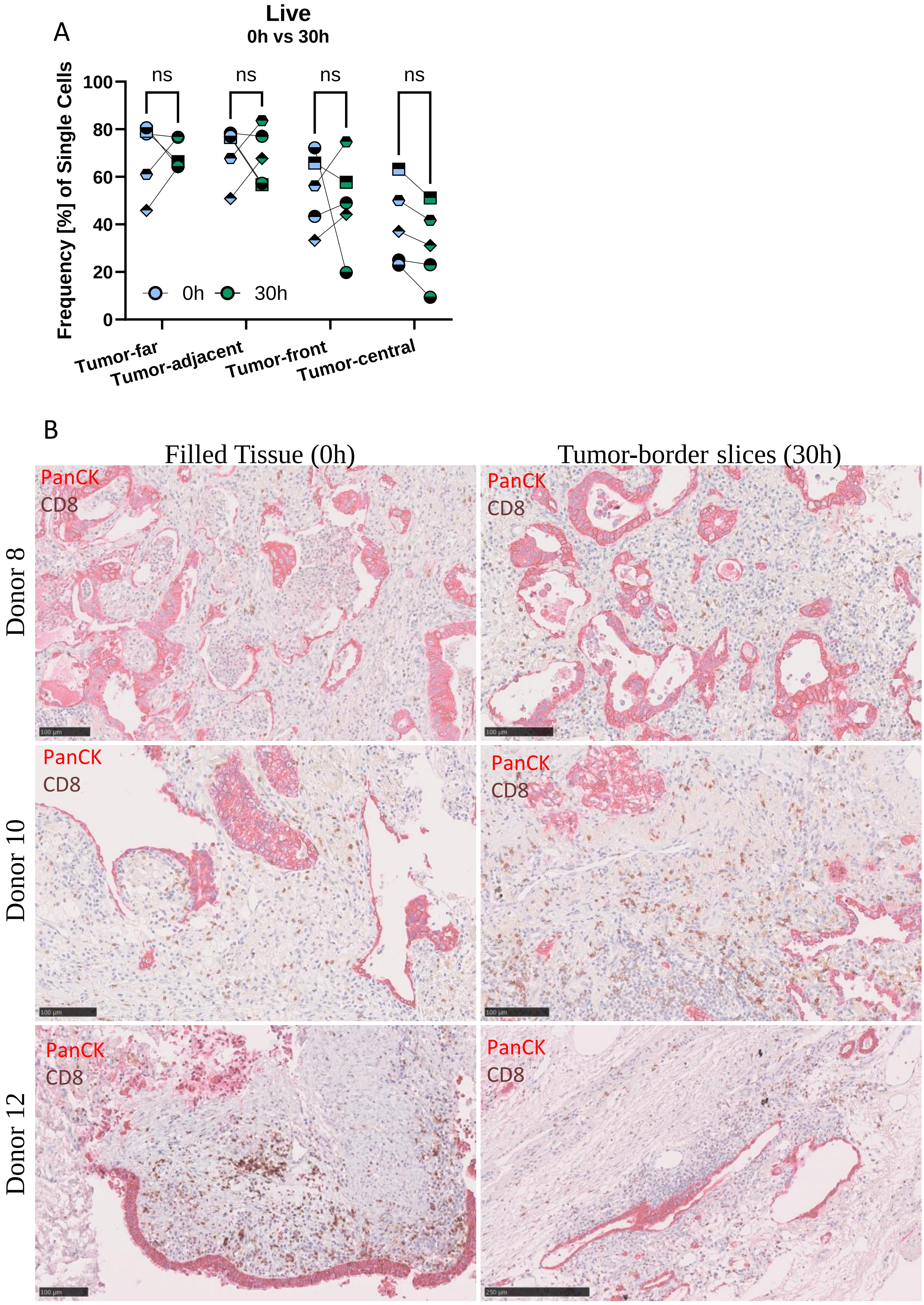

Supplementary Figure 6. Viability and histology remained unchanged after 30 hours of cultivation. *A) Frequency of live cells gated on single cells in tumor-far, tumor-adjacent, tumor-border, and tumor-central tissue before cultivation (0h) and after cultivation (30h) measured by flow cytometry. B) IHC staining of PanCK and CD8 in tumor-border tissue prior to cultivation (filled tissue) and post 30h cultivation (tumor-border slices).*

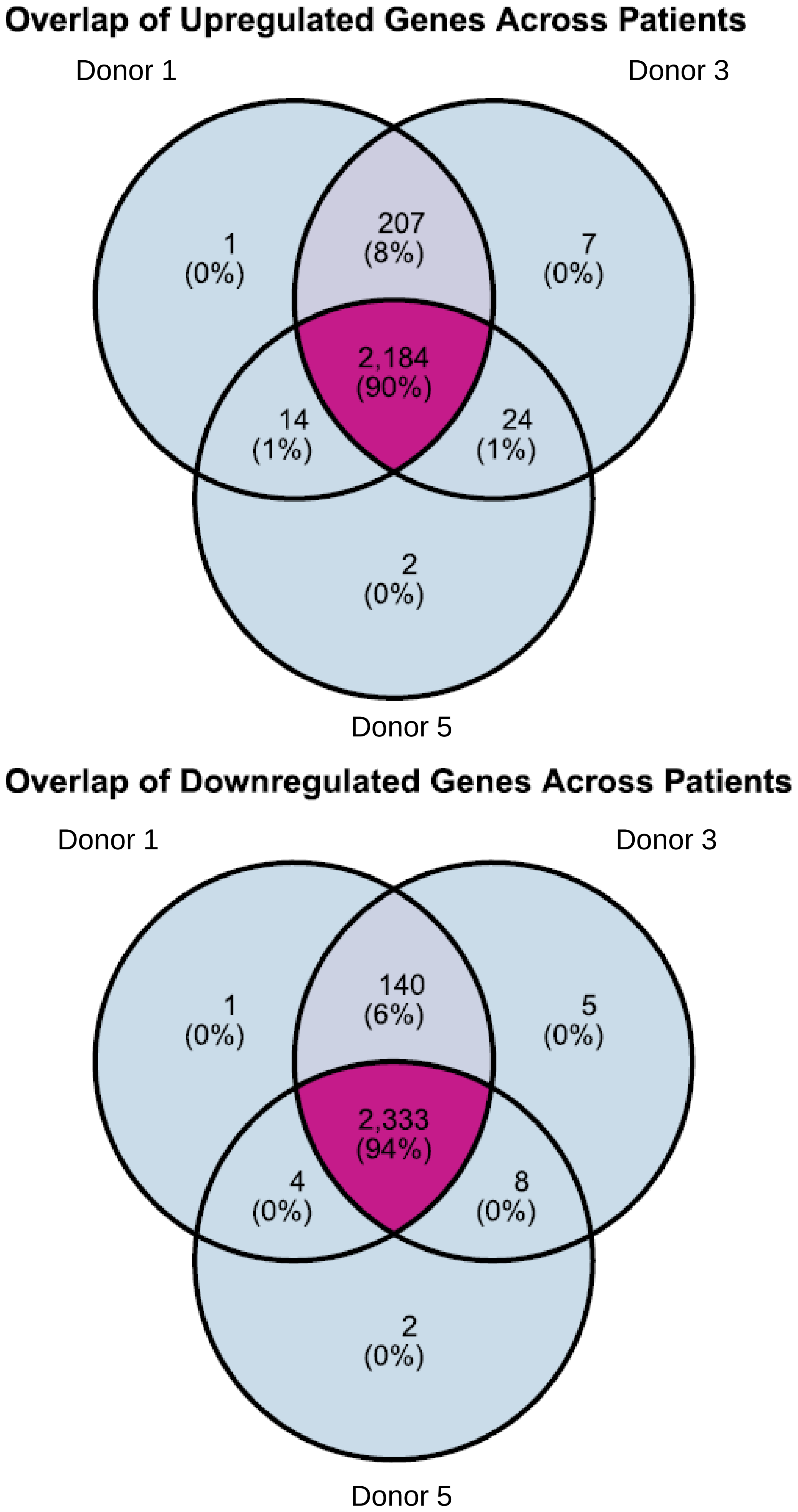

***Supplementary Figure 7. Overlap of differentially expressed genes across the three donors**. To assess the concordance of gene expression changes between donors, we evaluated the direction of regulation (up or down) for all differentially expressed genes (adjusted p-value < 0.05) identified in the overall RNA-seq analysis. This evaluation was performed in each individual donor using normalized count data comparing tumor-border tissue with non-tumor tissue. The Venn diagrams illustrate the overlap of: upregulated genes (n = 2,439), and downregulated genes (n = 2,493), as determined in the overall analysis and confirmed in each donor.*

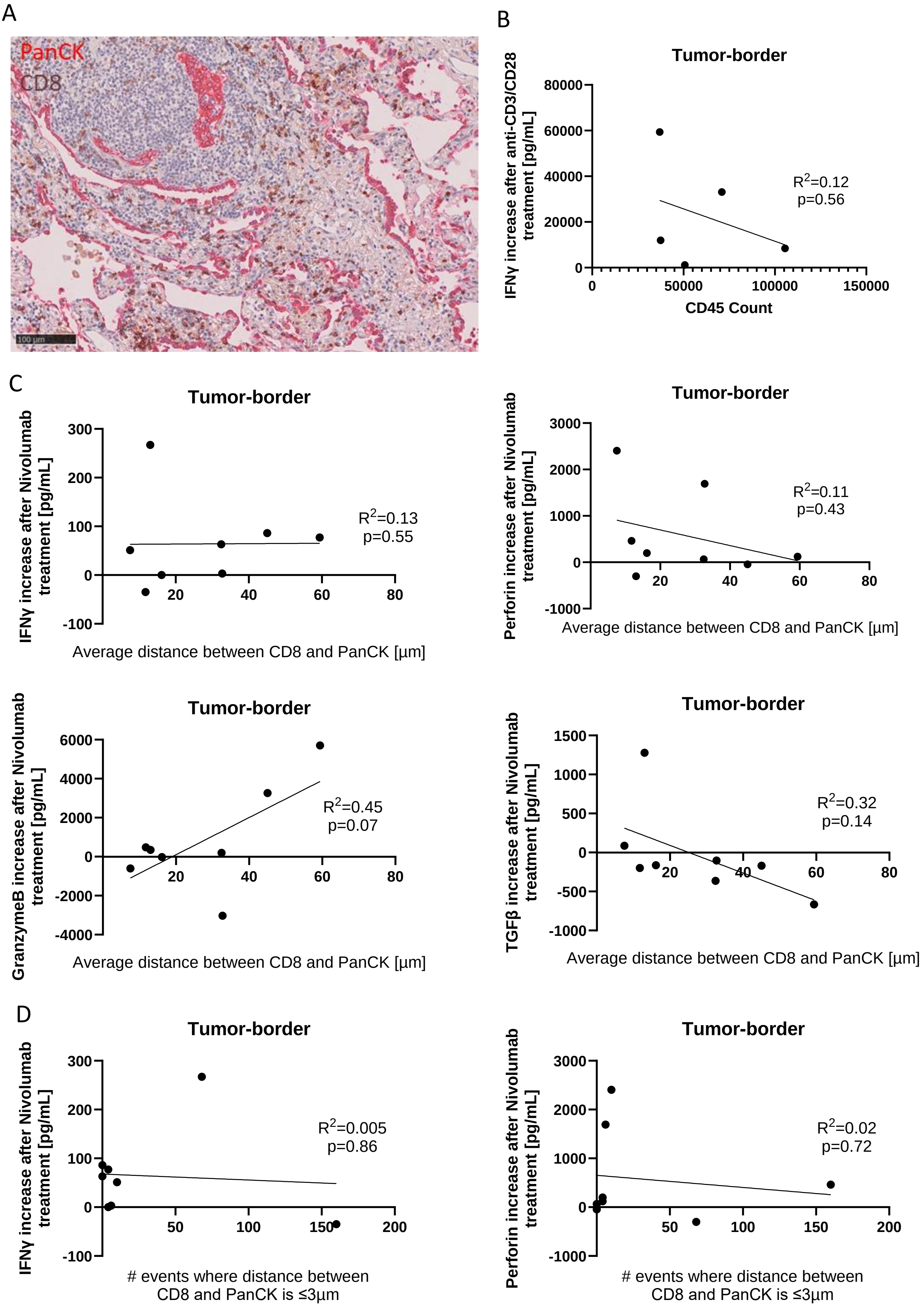

Supplementary Figure 8. Inflammatory cytokine secretion does not correlate with distance between PanCK expressing cells and $CD8^+$ T cells. *A) Sample immunofluoresence staining of $CD8^+$ and PanCK in a tumor-border slice. B) Increase of IFNγ after treatment with anti-CD3/CD28 compared to medium control (Y-axix) as measured in the supernatant of tissue slices versus average distance measured between $CD8^+$ and PanCK stained cells in immunoflouresence of matched samples (X-axis) at the tumor-border. C) Increase of IFNγ, Perforin, Granzyme B and decrase of TGFβ´after treatment with Nivolumab compared to medium control (Y-axix) as measured in the supernatant of tissue slices versus average distance measured between $CD8^+$ and PanCK stained cells in immunoflouresence of matched samples (X-axis) at the tumor-border. D) Change in cytokine level after treatment with Nivolumab compared to medium control (Y-axis) versus number of events where the distance between a $CD8^+$ T cell and a PanCK tumor cell is less than or equal to 3µm (Y-axis). C). R^2 and P-value for each linear regression correlation is depicted for n=5 or 8 donors.*

Supplementary Table 1. *Tumor type information linked to donors, including shapes as seen in all figures.*

Donor	Sex, Age	Tumor Type	Symbol
1	M, 54	Lung Adenocarcinoma	Circle
2	F, 76	Lung Adenocarcinoma	Hexagon
3	M, 76	Lung Adenocarcinoma	Half circle
4	W, 68	Lung Adenocarcinoma	Half hexagon
5	W, 64	Lung Adenocarcinoma	Square
6	M, 54	Lung Adenocarcinoma	Circle with X
7	W, 77	Metastase (Uterus, adenocarcinoma)	Half square
8	W, 67	Lung Adenocarcinoma	Triangle
9	M, 57	Metastasis (Colorectal Adenocarcinoma)	Sqare with x
10	W, 59	Lung Adenocarcinoma	Half Triangle
11	W, 69	Metastasis (cRCC)	Diamond
12	W, 54	Metastasis (MEC, salivary gland tumor)	Half diamond
13	M, 52	Lung Adenocarcinoma	Thick circle
14	W, 62	Lung Adenocarcinoma	Upsideown half triangle
15	M, 70	Lung Adenocarcinoma	Half circle right
16	M: 85	Lung Adenocarcinoma	Diamond with x
17	W, 61	Lung Adenocarcinoma	Hexagon with x
18	M, 85	Carcinoid Lung Tumor	Half triangle right

Supplementary Table 2. DEGs Non-tumor vs. tumor-border slices. *DEGs picked from complete list of DEGs based on high LogFC, low adjusted p-value and high relevance to tumor tissue.*

Gene	LogFC	Adjusted p-value
COL11A1	5.84158456	2E-06
MMP11	5.55387747	6E-15
S100P	2.75910655	2E-04
TNFRSF6B	2.4247068	8E-08
IL2RA	2.28448561	1E-03
ADAM8	2.1382023	3E-15
CD55	1.93550727	3E-08
CLDN2	1.83873707	9E-03
GSDMB	1.82543969	1E-07
LOXL2	1.61756074	1E-03
SRC	1.40439706	5E-08
FOXP4	1.07704092	1E-06
IFNGR2	1.00745524	2E-03
TGFB1	0.81679629	7E-03
IL6ST	-0.72360693	6E-03
S1PR1	-1.08835095	4E-03
SMAD1	-1.24309017	3E-04
CD163	-1.67145889	4E-04
IFIT1	-1.71011429	5E-04
CDH5	-1.72989848	1E-05
ICAM2	-1.90605667	2E-05
IL12A	-2.18668138	4E-05
CXCL6	-2.67276562	1E-04
SIGLEC1	-4.09012756	2E-06

Supplementary Table 3. Raw data of secreted factors in supernatant of donors 1-12.

Tissue Type	Soluble Factor	Donor 1	Donor 3	Donor 5	Donor 7	Donor 8	Donor 10	Donor 11	Donor 12
Non-tumor	CA15-3 [kU/L]				2	2	2	2	2
Tumor-border					9	2	2	3	3
Non-tumor	CA19-9 [kU/L]				4	3	3	3	3
Tumor-border					427	4494	3	3	62
Non-tumor	CA72-4 [kU/L]				2	2	2	2	2
Tumor-border					24	3	7	2	22
Non-tumor	CA125 [kU/L]				2	1	1	1	1
Tumor-border					369	4	3	1	200
Non-tumor	CEA [µg/L]				1	1	1	1	1
Tumor-border					2	6	204	1	2
Non-tumor	S100 [µg/L]				0.062	0.062	0.327	0.085	0.061
Tumor-border					0.367	0.367	2.57	0.959	2.32
Non-tumor	Ferritin [µg/L]				8	4	57	11	3
Tumor-border					871	420	741	736	383
Non-tumor	IL-6 [ng/L]				661	485	3766	578	1788
Tumor-border					27306	20795	50000	24262	54362
Non-tumor	IFNγ [pg/mL]	18.69	32.21	3.21	8.47	6.69	42.59	13.33	64.09
Tumor-border		15.20	17.49	8.34	71.86	59.28	387.05	39.45	193
Non-tumor	IL-2 [pg/mL]	166.58	132.35	15.05	11.52	0.00	33.25	0.00	21.84
Tumor-border		414.78	147.38	58.81	1220.09	92.78	154.92	59.50	166.03
Non-tumor	Granzyme B [pg/mL]	197.83	1296.51	314.50	25.41	7.47	11.52	17.56	32.34
Tumor-border		5433.97	3069.58	1559.72	391.02	4038.83	1220.09	892.51	271.47
Non-tumor	Perforin [pg/mL]	212.37	268.79	57.18	101.11	135.85	229.60	138.65	308.86
Tumor-border		371.65	85.18	71.65	276.15	2524.83	1329.36	2712.60	1142.08
Non-tumor	IL-17A [pg/mL]	1.15	1.47	0.88	3.62	4.07	37.92	0.70	5.33
Tumor-border		2.42	1.88	1.32	33.54	84.51	124.43	17.44	57.41
Non-tumor	IL-1β [pg/mL]	1.92	1.46	13.46	69.47	0.00	150.72	0.00	0.00
Tumor-border		87.93	7.01	17.73	4049.93	2212.83	5952.81	12644.91	2690.92
Non-tumor	TGFβ [pg/mL]	244.40	486.77	134.71	463.60	192.65	530.79	255.89	388.80
Tumor-border		2004.66	1174.58	893.80	3674.04	1954.67	2429.63	3284.15	2196.90
Non-tumor	IL10 [pg/mL]	49.92	19.62	643.57	1.95	0.82	2.76	1.28	1.66
Tumor-border		24.54	2.79	185.26	36.62	27.48	20.95	100.88	34.20

Supplementary Table 4. Raw data of secreted factors in supernatant of donors 13-18.

Tissue Type	Soluble Factor	Donor 13	Donor 14	Donor 15	Donor 16	Donor 17	Donor 18
Non-tumor	IFNγ [pg/mL]	7.79	33.83	13.17	11.73	26.01	35.41
Tumor-border		10.02	2.98	11.27	5.73	56.60	5.93
Non-tumor	IL-2 [pg/mL]	4.87	18.47	17.18	10.50	4.23	2.99
Tumor-border		97.12	10.11	11.67	13.75	32.59	14.84
Non-tumor	Granzyme B [pg/mL]	93.73	104.57	13.13	5.51	43.68	28.28
Tumor-border		73.84	547.43	7.95	9.75	1787.13	57.47
Non-tumor	Perforin [pg/mL]	189.72	678.03	134.97	128.00	153.84	70.44
Tumor-border		108.40	389.41	128.04	255.91	218.01	55.72
Non-tumor	IL-17A [pg/mL]	8.14	4.27	2.30	1.57	3.21	13.20
Tumor-border		5.07	48.23	1.74	1.93	15.44	4.62
Non-tumor	IL-1β [pg/mL]	0.31	0.55	2.71	0.48	4.39	23.87
Tumor-border		0.42	0.55	1.59	0.63	60.07	11.73
Non-tumor	TGFβ [pg/mL]	562.37	512.94	651.52	181.81	178.00	134.59
Tumor-border		720.49	292.98	566.19	206.82	414.89	186.48
Non-tumor	IL10 [pg/mL]	1.81	2.52	0.30	0.77	10.42	30.06
Tumor-border		0.49	10.62	0.28	0.78	10.25	2.41

Supplementary Table 5. IFNγ and TGFβ content in the supernatant, CD8$^+$ T cell count and frequency of CD8$^+$ T cells that express PD-1 in the tissue without any treatment. Fold-changes of IFNγ, Perforin and IL-2 after treatment with anti-CD3/CD28 or Nivolumab of non-tumor and tumor-border tissue.

Tissue Type	Parameter	Donor 7	Donor 8	Donor 10	Donor 11	Donor 12
Non-tumor	IFNγ [pg/mL] no treatment	8.48	6.69	42.59	13.33	64.09
Tumor-border		71.87	59.28	387.05	39.46	193.00
Non-tumor	TGFβ [pg/mL] no treatment	463.60	192.65	530.79	255.89	388.80
Tumor-border		3674.04	1954.67	2429.63	3284.15	2196.90
Non-tumor	CD8$^+$ T cell count	693	613	1445	1516	545
Tumor-border		4019	7482	12852	10092	4944
Non-tumor	PD-1 expresionon CD8$^+$ T cells	17.1	17.4	18.6	12.7	43.8
Tumor-border		43.6	28.6	21.6	50.2	31.7
**Anti-CD3/CD28**						
Non-tumor	TGFβ Fold-change	1.00	0.82	0.74	0.78	0.72
Tumor-border		1.04	0.52	1.10	1.11	0.65
Non-tumor	IFNγ Fold-change	529.77	2523.26	773.21	1455.62	336.04
Tumor-border		16.95	558.80	22.82	304.91	308.68
Non-tumor	IL-2 Fold-change	278.99	-	240.06	-	285.88
Tumor-border		2.87	837.71	30.94	54.32	491.98
Non-tumor	Granzyme B Fold-change	21.74	252.34	278.99	232.96	59.20
Tumor-border		2.39	11.30	2.87	5.59	202.44
**Nivolumab**						
Non-tumor	TGFβ Fold-change	1.16	1.87	1.96	1.05	0.80
Tumor-border		0.96	0.95	1.53	1.03	0.91
Non-tumor	IFNγ Fold-change	2.71	1.33	6.57	1.06	0.83
Tumor-border		1.00	1.05	1.69	2.28	0.82
Non-tumor	IL-2 Fold-change	6.27	-	1.06	-	0.62
Tumor-border		1.28	1.23	1.43	1.98	0.94
Non-tumor	Perforin Fold-change	0.90	1.82	4.46	0.88	0.84
Tumor-border		1.71	1.67	0.77	1.89	1.41
